# Long COVID is associated with female sex; Anti-NCAM1 autoantibodies are absent in patients with long COVID

**DOI:** 10.1016/j.ibneur.2025.07.002

**Published:** 2025-07-09

**Authors:** Yukiko Motokawa, Jun Sugihara, Tomoya Tateishi, Tadashi Hosoya, Shinsuke Yasuda, Yasunari Miyazaki, Hidehiko Takahashi, Hiroki Shiwaku

**Affiliations:** a Department of Psychiatry and Behavioral Sciences, Graduate School of Medical and Dental Sciences, Institute of Science Tokyo, Tokyo, Japan; b Department of Respiratory Medicine, Graduate School of Medical and Dental Sciences, Institute of Science Tokyo, Tokyo, Japan; c Department of Rheumatology, Graduate School of Medical and Dental Sciences, Institute of Science Tokyo, Tokyo, Japan

**Keywords:** Long COVID, Anti-NCAM1 autoantibody, NCAM1, Autoantibody, SARS-CoV-2

## Abstract

**Background:**

Long COVID is a condition that may arise following SARS-CoV-2 infection and is associated with a range of systemic complications. Autoantibodies are implicated in the pathogenesis of long COVID. However, the details of the pathogenic mechanisms undergone by these autoantibodies remain unclear. Neural cell adhesion molecule 1 (NCAM1) is the human protein with the highest sequence homology to the SARS-CoV-2 proteins. Previous in silico studies indicate that SARS-CoV-2 infection may induce the production of anti-NCAM1 autoantibodies. Thus, this study investigated the presence of anti-NCAM1 autoantibodies in individuals affected by COVID-19, including those with long COVID.

**Methods:**

Serum samples were obtained from 173 individuals 3 months after SARS-CoV-2 infection. Among them, 63 were diagnosed with long COVID. A cell-based assay was used to assess all 173 serum samples for the presence of anti-NCAM1 autoantibodies. We also analyzed the clinical profiles of patients with and without long COVID to identify potential risk factors associated with long COVID.

**Results:**

Anti-NCAM1 autoantibodies were not detected in any serum sample. The proportion of female patients in the long COVID group was significantly higher than that in the non-long COVID group.

**Conclusion:**

The results indicate that the production of anti-NCAM1 autoantibodies following COVID-19 is unlikely. Female sex is associated with higher risk of long COVID.

## Introduction

COVID-19 is an infectious disease caused by the SARS-CoV-2 virus that may result in several systemic complications and long-term effects. When such symptoms persist beyond the acute phase, the condition is referred to as long COVID (Ely et al., 2024, Lopez-Leon et al., 2021, Thaweethai et al., 2023). The clinical presentation of long COVID varies, but fatigue, shortness of breath, and cognitive dysfunction are common symptoms experienced by patients (Ely and Brown, 2024; Thaweethai et al., 2023).

Long COVID is defined as the persistence of symptoms for at least 2 months after the initial SARS-CoV-2 infection, with symptoms that are either new or unresolved and not attributable to other diagnoses (Soriano et al., 2022). Long COVID is typically diagnosed 3 months after the onset of acute COVID-19 (Soriano et al., 2022). This syndrome affects multiple organ systems, particularly the nervous and vascular systems (Monje and Iwasaki, 2022, Xu et al., 2022). The proposed pathological mechanism involves the production of autoantibodies in response to SARS-CoV-2 infection (Davis et al., 2023, Franke et al., 2021, Jernbom et al., 2024, Wang et al., 2021).

A potential trigger for the production of these autoantibodies is molecular mimicry, a process by which the immune system produces antibodies that cross-react with host tissues (Cusick et al., 2012, Rojas et al., 2018, Smatti et al., 2019). Sequence analysis of SARS-CoV-2 components identified neural cell adhesion molecule 1 (NCAM1) as the protein with the highest similarity to an envelope protein of SARS-CoV-2 (Morsy, 2020). These findings raise the hypothesis that SARS-CoV-2 infection may induce the production of anti-NCAM1 autoantibodies (Morsy, 2020).

NCAM1 is a cell adhesion molecule that is predominantly expressed in the nervous system, where it localizes to both presynaptic and postsynaptic sites, mediating synapse formation through homophilic interactions (Duncan et al., 2021, Sytnyk et al., 2017). In addition to its membrane-bound form, NCAM1 circulates in a soluble form and is found in various tissues, including the kidney and muscles (Caza et al., 2021, Illa et al., 1992).

Anti-NCAM1 autoantibodies have been detected in patients with schizophrenia and membranous lupus nephritis (Caza et al., 2021; Shiwaku et al., 2022). Anti-NCAM1 autoantibodies reduce synaptic density and impair cognitive function (Shiwaku et al., 2022). Furthermore, a case report has described a patient with encephalitis and membranous lupus nephritis who was seropositive for anti-NCAM1 autoantibodies (Caza et al., 2021). However, the presence of anti-NCAM1 autoantibodies in individuals following COVID-19 has not yet been investigated. Moreover, other potential risk factors associated with long COVID remain insufficiently characterized.

Thus, this study investigated the prevalence of anti-NCAM1 autoantibodies in patients following SARS-CoV-2 infection. We analyzed serum samples from 173 patients that were collected 3 months after COVID-19 using a cell-based assay that had been previously validated for the detection of anti-NCAM1 autoantibodies. We also analyzed the clinical characteristics of patients with and without long COVID to identify potential risk factors associated with long COVID.

## Methods

### Participants and inclusion/exclusion criteria

Serum samples were collected from 173 patients 3 months after the confirmed SARS-CoV-2 infection. The enrolled patients were hospitalized at the Institute of Science Tokyo Hospital for COVID-19 treatment between November 15, 2020, and September 5, 2021, and subsequently followed up as outpatients. COVID-19 was diagnosed based on positive reverse transcription polymerase chain reaction results for SARS-CoV-2. Patients aged < 20 years were excluded. Long COVID was diagnosed based on the previously reported criteria (Soriano et al., 2022).

### Ethics

This study was performed in strict accordance with the Ethical Guidelines for Medical and Health Research Involving Human Subjects in Japan. It was approved by the Committees on Gene Recombination Experiments and Human Ethics of the Institute of Science Tokyo (G2025–008A and G2020–006). All participants provided written informed consent.

### Clinical data collection

Data on clinical outcomes, including sex, age, height, weight, smoking status, comorbidities at presentation, severity of acute respiratory failure, medications used during the acute phase, and residual or new COVID-19 symptoms 3 months after diagnosis, were collected from electronic medical records. Laboratory tests were conducted at the 3-month follow-up visit, including blood tests such as white blood cell differential, C-reactive protein, ferritin, and D-dimer.

### Cell culture and transfection

HeLa cells were cultured in 5 % CO2 in Dulbecco's Modified Eagle Medium (SIGMA, MI, USA), containing 10 % fetal bovine serum (FBS), at 37 °C. For transfection, cells were treated with plasmids using Lipofectamine 2000 (Thermo Fisher Scientific, Waltham, MA, USA), following the guidelines provided by the manufacturer.

### Plasmids

NCAM1 and enhanced green fluorescent protein (EGFP) were cloned into a pRP vector (VectorBuilder) and expressed via the cytomegalovirus (CMV) promoter, as previously described (Shiwaku et al., 2022).

### Cell-based assays and Immunocytochemistry

A cell-based assay was performed using HeLa cells with an immunocytochemical approach to detect anti-NCAM1 autoantibodies. HeLa cells were fixed using 2 % paraformaldehyde (dissolved in phosphate-buffered saline [PBS]) at room temperature for 30 min and then treated with 0.1 % Triton X-100 in PBS for about 10 min. Afterward, the cells were blocked for 30 min at room temperature using PBS with 10 % FBS and then incubated with serum or a primary antibody diluted in a blocking buffer. Immunocytochemistry was performed using anti-NCAM1 (1:200, MA5–11563, Thermo Scientific). It was detected using Cy3-conjugated anti-mouse IgG (1:500, 715-165-150, Jackson Laboratory), and serum autoantibodies were detected using Cy3-conjugated anti-human IgG (1:500, 709-165-149, Jackson Laboratory, Bar Harbor, ME, USA). Nuclei were stained with DAPI (0.2 μg/mL in PBS; DOJINDO). Images were acquired under an Olympus FV1200 confocal microscope (Tokyo, Japan). Serum with an autoantibody titer of ≥ 1:30 was considered positive for autoantibodies, as previously described (Shiwaku et al., 2023, Shiwaku et al., 2020; Shiwaku et al., 2022).

### Statistical analysis

Categorical variables are expressed as numbers with percentages, while continuous variables are expressed as medians with interquartile ranges. Statistical analyses were conducted with R version 4.3.2. as follows: Fisher exact test (for categorical variables) or Wilcoxon rank sum test (for continuous variables). *p*-values < 0.05 were considered to indicate statistical significance.

## Results

**Fig. 1:**
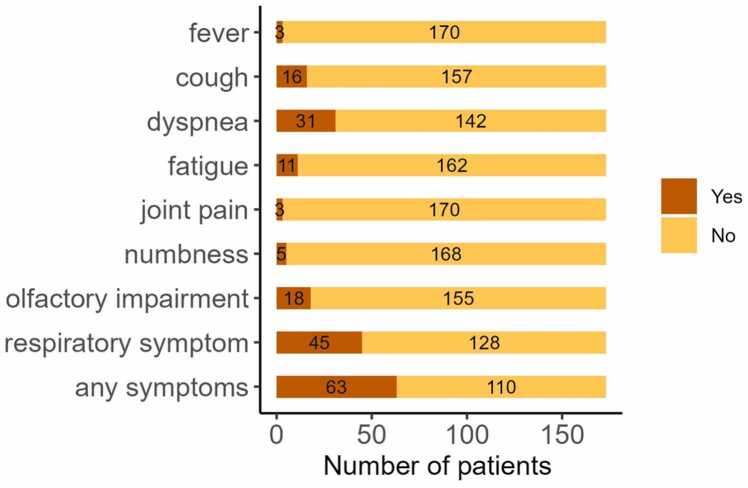
Number of patients with residual symptoms.

Serum samples were collected from 173 patients 3 months after diagnosis of SARS-CoV-2 infection. At the 3-month follow-up, 63 of the 173 patients reported persistent clinical symptoms, such as respiratory complaints or fatigue, and met the diagnostic criteria for long COVID (Fig. 1, Table 1). The remaining 110 patients, who reported no symptoms, were classified as not having long COVID (Fig. 1, Table 1).

**Table 1:** Patient characteristics.

	All	Long COVID (+)	Long COVID (-)	*p* value
Total number of patients	173	63	110	
Sex (female)	63 (36.4 %)	29 (46.0 %)	34 (30.9)	< 0.0001
Age (years)	55 [47–67]	55 [47–68]	55.5 [46–66]	0.8092
(sixty-five or more)	59 (30.3 %)			
BMI	24.4 [22.4–27.25]	24.7 [22.6–27.2]	24.25 [22.4–27.3]	0.5985
Comorbidity				
Cardiovascular	22 (12.7 %)	5 (7.9 %)	17 (15.5 %)	0.2350
Hypertension	60 (34.7 %)	23 (36.5 %)	37 (33.6 %)	0.7414
Diabetes mellitus	28 (16.2 %)	10 (15.9 %)	18 (16.4 %)	1.0000
Malignancy (active)	7 (4.0 %)	2 (3.2 %)	5 (4.5 %)	1.0000
Malignancy (in remission)	11 (6.4 %)	5 (7.9 %)	6 (5.5 %)	0.5318
Autoimmune disease	17 (9.8 %)	7 (11.1 %)	10 (9.1 %)	0.7915
Respiratory	26 (15.0 %)	13 (20.6 %)	13 (11.8 %)	0.1275
Smoking status at admission				
Never smoker	78 (45.1 %)	23 (36.5 %)	55 (50.0 %)	0.1293
Ex-smoker	65 (37.6 %)	25 (39.7 %)	40 (36.4 %)	
Current smoker	23 (13.3 %)	12 (19.0 %)	11 (10.0 %)	
Respiratory failure in the acute phase				
None	64 (37.0 %)	21 (33.3 %)	43 (39.1 %)	0.6871
Oxygen	92 (53.2 %)	34 (54.0 %)	58 (52.7 %)	
Intubation	16 (9.2 %)	7 (11.1 %)	9 (8.2 %)	
Treatment				
Remdesivir	91 (52.9 %)	32 (51.6 %)	59 (53.6 %)	0.8739
Corticosteroid	99 (57.6 %)	36 (58.1 %)	63 (57.3 %)	1.0000
Tocilizumab	10 (5.8 %)	4 (6.5 %)	6 (5.5 %)	0.7484
Baricitinib	22 (12.8 %)	10 (16.1 %)	12 (10.9 %)	0.3482

The proportion of female patients in the long COVID-19 (+) group was significantly higher than that in the long COVID-19 (–) group (Table 1). However, no significant differences were observed between the two groups in terms of age, body weight, comorbidities, smoking status, respiratory failure during the acute phase, or COVID-19 treatment (Table 1). Furthermore, no significant differences were observed in the 3-month follow-up blood test results between the two groups (supplemental Table 1). Given the sample size, the study had sufficient power (>80 %) to detect moderate group differences (effect size h ≈ 0.4), suggesting that the lack of significant findings reflects true absence of clinically meaningful differences rather than insufficient power.

**Fig. 2:**
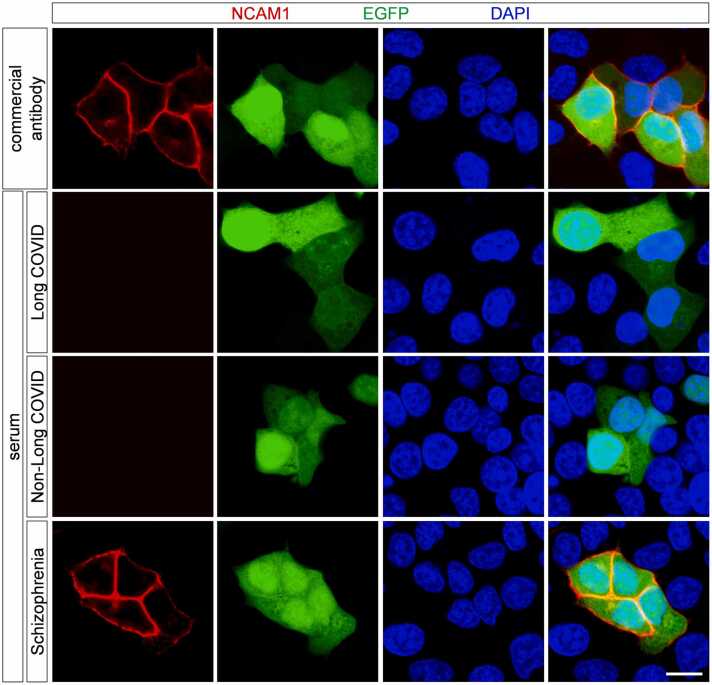
Anti-NCAM1 autoantibodies are absent in patients with long COVID. A cell-based assay was performed using the serum of patients with long COVID, non-long COVID and anti-NCAM1 autoantibody-positive schizophrenia. Immunocytochemistry was performed using a commercially available anti-NCAM1 antibody. Scale bar: 10 μm.

Anti-NCAM1 autoantibodies were detected using a cell-based assay. Briefly, HeLa cells were transfected with a plasmid co-expressing EGFP (as a transfection marker) and NCAM1, and analyzed by immunocytochemistry. None of the 173 samples tested positive for anti-NCAM1 autoantibodies (Fig. 2).

Commercially available anti-NCAM1 antibodies and serum samples from patients with schizophrenia previously confirmed to be anti-NCAM1 seropositive were used as positive controls. Anti-NCAM1 autoantibodies were successfully detected in both sample types (Fig. 2).

## Discussion

In this study, we found female sex is associated with higher risk of long COVID. However, no anti-NCAM1 autoantibodies were detected in any of the serum samples collected 3 months after the COVID-19 diagnosis.

Although a previous in silico study has hypothesized that SARS-CoV-2 infection can trigger the production of anti-NCAM1 autoantibodies, (Morsy, 2020) our findings indicate that if autoantibodies are indeed produced, the occurrence is rare. Although we did not detect anti-NCAM1 autoantibodies associated with COVID-19, our results verify their specificity in diseases such as schizophrenia and membranous lupus nephritis, where their presence has been previously established (Caza et al., 2021; Shiwaku et al., 2022)

The characteristics of the current study cohort align with those in earlier reports indicating a higher prevalence of long COVID in female patients compared with non-Long COVID cohort (Bai et al., 2022, Shah et al., 2025). Although the association between female sex and long COVID was not the primary objective of the present study, the replication of this pattern in an independent cohort reinforces its potential biological significance. A notable strength of our cohort is that it included patients with confirmed acute COVID-19 who were followed prospectively for 3 months, reducing potential selection bias related to sex-specific recruitment.

The mechanisms linking female sex and long COVID remain unclear, but several plausible hypotheses exist. For instance, chronic fatigue syndrome (CFS), which shares clinical features with long COVID, is more prevalent among women (Davis et al., 2023; Pollack et al., 2023). Both conditions are associated with autoimmune processes, and autoimmune disorders are more common in women (Desai and Brinton, 2019, Dou et al., 2024; Jernbom et al., 2024; Sotzny et al., 2018; Wang et al., 2021). Therefore, autoimmune mechanisms and CFS may serve as shared pathological pathways connecting female sex and long COVID. Additionally, estrogen—known to modulate the immune, central nervous, and endocrine systems—may contribute to sex-based differences in susceptibility (Peluso and Deeks, 2024). Nonetheless, the underlying biological mechanisms remain to be elucidated, warranting further investigation.

This study has several limitations. First, the number of long COVID cases included was relatively small. A larger sample size may include rare cases in which anti-NCAM1 autoantibodies may be detected. However, because the 173 individuals included in the cohort were sampled after SARS-CoV-2 infection, anti-NCAM1 autoantibodies—if produced at any significant frequency—would likely have been detected, even at low titers. The negative results indicate that their prevalence after COVID-19 is likely to be low. Second, detailed assessments of cognitive or psychiatric symptoms were not conducted. Thus, possibly, some patients may have had such symptoms but were not identified; thus, the actual percentage of long COVID cases may have been underestimated. Nonetheless, even if additional cases were classified as long COVID, our conclusion remains that anti-NCAM1 autoantibodies are not commonly associated with COVID-19. Because anti-NCAM1 autoantibodies have been linked to cognitive and psychiatric dysfunction, we cannot fully exclude the possibility that our cohort included relatively few individuals with severe neuropsychiatric symptoms. However, cognitive dysfunction is a common feature of long COVID; thus, this scenario is unlikely (Thaweethai et al., 2023). Further studies assessing patients with long COVID and concurrent marked cognitive or psychiatric symptoms, especially those severe enough to warrant psychiatric clinical intervention, may uncover deeper insights into the potential involvement of anti-NCAM1 autoantibodies in patients with long COVID.

A previous study involving a small group of patients exhibiting prominent neurological symptoms reported frequent presence of autoantibodies targeting neural molecules (Franke et al., 2021). Therefore, assessing the presence of anti-NCAM1 autoantibodies in these cases is also critical for future research. In this context, it is important to ask whether our cohort included individuals with autoantibodies to begin with. However, prior studies have indicated that long COVID is not always accompanied by elevated inflammatory marker levels, which limits the usefulness of standard blood tests for predicting autoantibody presence (Erlandson et al., 2024). Consistently, our cohort showed no significant changes in C-reactive protein (CRP) levels or white blood cell counts. Furthermore, although NCAM1 and other neuronal autoantibodies have been detected in individuals with psychiatric disorders, they lack systemic inflammatory responses as well (Katayama et al., 2025, [Bibr bib14][Bibr bib23], Shiwaku et al., 2022 Shiwaku et al., 2020). This underscores the importance of directly measuring the autoantibodies, as was done in the present study. Additionally, antiphospholipid antibodies were identified in approximately 40 % of acute COVID patients admitted to the same hospital during a similar time frame, supporting the plausibility of autoantibody production in our cohort (Oba et al., 2023).

In conclusion, anti-NCAM1 autoantibodies were not detected in the serum samples collected from patients 3 months after diagnosis with COVID-19. However, female sex is associated with higher risk of long COVID. Further studies are warranted to better understand the etiologies underlying long COVID.

## CRediT authorship contribution statement

**Jun Sugihara:** Writing – review & editing, Validation, Software, Resources, Methodology, Investigation, Formal analysis, Data curation, Conceptualization. **Tomoya Tateishi:** Writing – review & editing, Validation, Resources, Funding acquisition, Conceptualization. **Tadashi Hosoya:** Writing – review & editing, Validation, Supervision, Resources, Conceptualization. **Shinsuke Yasuda:** Writing – review & editing, Validation, Supervision, Resources. **Yasunari Miyazaki:** Writing – review & editing, Validation, Supervision, Resources. **Hidehiko Takahashi:** Writing – review & editing, Validation, Supervision. **Hiroki Shiwaku:** Writing – review & editing, Writing – original draft, Visualization, Validation, Supervision, Software, Resources, Project administration, Methodology, Investigation, Funding acquisition, Formal analysis, Data curation, Conceptualization. **Yukiko Motokawa:** Writing – review & editing, Writing – original draft, Visualization, Validation, Resources, Methodology, Investigation, Formal analysis, Data curation.

## Declaration of Competing Interest

The authors declare that they have no known competing financial interests or personal relationships that could have appeared to influence the work reported in this paper.

## References

[bib1] Bai F., Tomasoni D., Falcinella C., Barbanotti D., Castoldi R., Mulè G. (2022). Female gender is associated with long COVID syndrome: a prospective cohort study. Clin. Microbiol. Infect..

[bib2] Caza T.N., Hassen S.I., Kuperman M., Sharma S.G., Dvanajscak Z., Arthur J. (2021). Neural cell adhesion molecule 1 is a novel autoantigen in membranous lupus nephritis. Kidney Int..

[bib3] Cusick M.F., Libbey J.E., Fujinami R.S. (2012). Molecular mimicry as a mechanism of autoimmune disease. Clin. Rev. Allergy Immunol..

[bib4] Davis H.E., McCorkell L., Vogel J.M., Topol E.J. (2023). Long COVID: major findings, mechanisms and recommendations. Nat. Rev. Microbiol..

[bib5] Desai M.K., Brinton R.D. (2019). Autoimmune disease in women: endocrine transition and risk across the lifespan. Front Endocrinol. (Lausanne).

[bib6] Dou D.R., Zhao Y., Belk J.A., Zhao Y., Casey K.M., Chen D.C. (2024). Xist ribonucleoproteins promote female sex-biased autoimmunity. Cell.

[bib7] Duncan B.W., Murphy K.E., Maness P.F. (2021). Molecular mechanisms of L1 and NCAM adhesion molecules in synaptic pruning, plasticity, and stabilization. Front. Cell. Dev. Biol..

[bib8] Ely E.W., Brown L.M., Fineberg H.V. (2024). Long Covid Defined. N. Engl. J. Med..

[bib9] Erlandson K.M., Geng L.N., Selvaggi C.A., Thaweethai T., Chen P., Erdmann N.B. (2024). Differentiation of Prior SARS-CoV-2 Infection and Postacute Sequelae by Standard Clinical Laboratory Measurements in the RECOVER Cohort. Ann. Intern. Med..

[bib10] Franke C., Ferse C., Kreye J., Reincke S.M., Sanchez-Sendin E., Rocco A. (2021). High frequency of cerebrospinal fluid autoantibodies in COVID-19 patients with neurological symptoms. Brain Behav. Immun..

[bib11] Illa I., Leon-Monzon M., Dalakas M.C. (1992). Regenerating and denervated human muscle fibers and satellite cells express neural cell adhesion molecule recognized by monoclonal antibodies to natural killer cells. Ann. Neurol..

[bib12] Jernbom A.F., Skoglund L., Pin E., Sjöberg R., Tegel H., Hober S. (2024). Prevalent and persistent new-onset autoantibodies in mild to severe COVID-19. Nat. Commun..

[bib13] Katayama S., Nayanar G., Suga T., Watanabe M., Takao C., Umezaki Y. (2025). Prevalence of the anti-CASPR2 autoantibody in patients with somatic symptom disorder accompanied by medically unexplained pain. Brain Behav. Immun. Health.

[bib14] Lennox B.R., Palmer-Cooper E.C., Pollak T., Hainsworth J., Marks J., Jacobson L. (2017). Prevalence and clinical characteristics of serum neuronal cell surface antibodies in first-episode psychosis: a case-control study. Lancet Psychiatry.

[bib15] Lopez-Leon S., Wegman-Ostrosky T., Perelman C., Sepulveda R., Rebolledo P.A., Cuapio A. (2021). More than 50 long-term effects of COVID-19: a systematic review and meta-analysis. Sci. Rep..

[bib16] Monje M., Iwasaki A. (2022). The neurobiology of long COVID. Neuron.

[bib17] Morsy S. (2020). NCAM protein and SARS-COV-2 surface proteins: In-silico hypothetical evidence for the immunopathogenesis of Guillain-Barré syndrome. Med. Hypotheses.

[bib18] Oba S., Hosoya T., Kaneshige R., Kawata D., Yamaguchi T., Mitsumura T. (2023). Thrombosis and antiphospholipid antibodies in Japanese COVID-19: based on propensity score matching. Front. Immunol..

[bib19] Peluso M.J., Deeks S.G. (2024). Mechanisms of long COVID and the path toward therapeutics. Cell.

[bib20] Pollack B., von, Saltza E., McCorkell L., Santos L., Hultman A., Cohen A.K. (2023). Female reproductive health impacts of Long COVID and associated illnesses including ME/CFS, POTS, and connective tissue disorders: a literature review. Front. Rehabil. Sci..

[bib21] Rojas M., Restrepo-Jiménez P., Monsalve D.M., Pacheco Y., Acosta-Ampudia Y., Ramírez-Santana C. (2018). Molecular mimicry and autoimmunity. J. Autoimmun..

[bib22] Shah D.P., Thaweethai T., Karlson E.W., Bonilla H., Horne B.D., Mullington J.M. (2025). Sex differences in long COVID. JAMA Netw. Open.

[bib23] Shiwaku H., Katayama S., Gao M., Kondo K., Nakano Y., Motokawa Y. (2023). Analyzing schizophrenia-related phenotypes in mice caused by autoantibodies against NRXN1α in schizophrenia. Brain Behav. Immun..

[bib24] Shiwaku H., Katayama S., Kondo K., Nakano Y., Tanaka H., Yoshioka Y. (2022). Autoantibodies against NCAM1 from patients with schizophrenia cause schizophrenia-related behavior and changes in synapses in mice. Cell Rep. Med..

[bib25] Shiwaku H., Nakano Y., Kato M., Takahashi H. (2020). Detection of autoantibodies against GABA(A)Rα1 in patients with schizophrenia. Schizophr. Res..

[bib26] Smatti M.K., Cyprian F.S., Nasrallah G.K., Al Thani A.A., Almishal R.O., Yassine H.M. (2019). Viruses and autoimmunity: a review on the potential interaction and molecular mechanisms. Viruses.

[bib27] Soriano J.B., Murthy S., Marshall J.C., Relan P., Diaz J.V. (2022). A clinical case definition of post-COVID-19 condition by a Delphi consensus. Lancet Infect. Dis..

[bib28] Sotzny F., Blanco J., Capelli E., Castro-Marrero J., Steiner S., Murovska M. (2018). Myalgic encephalomyelitis/chronic fatigue syndrome - evidence for an autoimmune disease. Autoimmun. Rev..

[bib29] Sytnyk V., Leshchyns'ka I., Schachner M. (2017). Neural cell adhesion molecules of the immunoglobulin superfamily regulate synapse formation, maintenance, and function. Trends Neurosci..

[bib30] Thaweethai T., Jolley S.E., Karlson E.W., Levitan E.B., Levy B., McComsey G.A. (2023). Development of a Definition of Postacute Sequelae of SARS-CoV-2 Infection. JAMA.

[bib31] Wang E.Y., Mao T., Klein J., Dai Y., Huck J.D., Jaycox J.R. (2021). Diverse functional autoantibodies in patients with COVID-19. Nature.

[bib32] Xu E., Xie Y., Al-Aly Z. (2022). Long-term neurologic outcomes of COVID-19. Nat. Med..

