# Supplementary material for: Long COVID is associated with female sex; Anti-NCAM1 autoantibodies are absent in patients with long COVID
Source: IBRO Neurosci Rep. 2025 Jul 9;19:252–6. doi: 10.1016/j.ibneur.2025.07.002 (PMC12275955; doi:10.1016/j.ibneur.2025.07.002)
Supplement: Supplementary file 1 — Supplementary material [file mmc1.docx]

|  |  | All | Long COVID (+) | Long COVID (-) | p value |
| --- | --- | --- | --- | --- | --- |
| WBCs | (x10^9^/L) | 5.80 [5.00–7.10] | 6.00 [5.10–7.00] | 5.75 [4.80–7.50] | 0.3029 |
| Neutrophils | (x10^9^/L) | 3.31 [2.60–4.29] | 3.47 [2.76–4.39] | 3.22 [2.53–4.20] | 0.2682 |
| Lymphocytes | (x10^9^/L) | 1.91 [1.46–2.35] | 1.97 [1.47–2.26] | 1.90 [1.46–2.39] | 0.9736 |
| Hemoglobin | (g/L) | 143 [132–153] | 143 [131–154] | 144 [133–153] | 0.7704 |
| Platelets | (x10^9^/L) | 248 [216–283] | 246 [216–294] | 249 [217–277] | 0.5087 |
| CRP | (mg/L) | 0.70 [0.30–1.60] | 0.60 [0.40–1.75] | 0.70 [0.30–1.60] | 0.7878 |
| Ferritin | (ng/ml) | 129 [61–215] | 149 [64–223] | 126 [58–206] | 0.6394 |
| D-dimer | (mg/L) | 0.50 [0.50–0.50] | 0.50 [0.50–0.50] | 0.50 [0.50–0.50] | 0.4589 |

* Abbreviations: WBC, white blood cell; CRP, C-reactive protein.

Supplemental Table 1. 3-month follow-up blood test data
